# Management of Psychosis in the Setting of Binge Eating, Mania, and Extrapyramidal Side Effects

**DOI:** 10.7759/cureus.26846

**Published:** 2022-07-14

**Authors:** Jean C Tamayo Acosta, Ariel Sosa Gomez, Leonel Gonzalez Diaz, Grecia Rivera Rodriguez, Marjorie Acosta Guillot

**Affiliations:** 1 Psychiatry, University of Medicine and Health Sciences, Bayamon, PRI; 2 Psychiatry, Saint James School of Medicine, Miami, USA; 3 Psychiatry, Ross University School of Medicine, Miami, USA; 4 Psychiatry, University of Puerto Rico, Medical Sciences Campus, Bayamon, PRI; 5 Psychiatry, Hospital Pavia Hato Rey, Bayamon, PRI

**Keywords:** antipsychotic management, psychomotor agitation, polypharmacy, tardive dyskinesia, extrapyramidal side effects, echolalia, hyponatremia, psychiatric management, eating disorder, psychosis

## Abstract

Psychosis is a mental disorder in which an individual exhibits a loss of contact with reality; this definition, however, often fails to convey the broadness and complexity behind the diagnosis. While studies agree that it is best practice to address psychosis by treating its underlying cause, manifestations of psychosis do vary widely and may be challenging to identify in some clinical scenarios, such as the one presented here. Binge eating in the setting of psychosis has been observed in instances where alterations of the gut microbiota in response to an eating disorder trigger psychotic episodes. However, instances in which the manifestation of psychosis itself is the catalytic factor for the presentation of a binge-eating event with aggression and delusions are seldom observed in the current medical literature. Of note, many of the drugs used to treat mental illness have been associated with regulating food intake. We aim to further expand on the association between psychosis, eating disorders, and management thereof in the setting of polypharmacy and undesired side effects. Here, we present the case and management of a 71-year-old male Hispanic patient with a significant history of mental illness who was admitted to the hospital due to acute gastroenteritis precipitated by binge eating during a psychotic episode.

## Introduction

Psychosis is a condition of the mind broadly defined as a loss of contact with reality [1]. Psychotic symptoms can increase patients’ risk of harming themselves or others or being unable to meet their basic needs. While it may be observed in many psychiatric disorders, it is often found in schizophrenia and mood disorders such as bipolar disorder and major depressive disorder with psychotic features. It may also be a manifestation of substance abuse or underlying medical disease. The broadness of the diagnosis is often the cause of its complexity, especially when it comes to stabilization of the patient, as failure to treat may cost the individual their life or even place others in harm’s way. “The incidence of psychosis worldwide has been estimated at approximately 50 in 100,000 people, while the incidence of schizophrenia (the most frequent eventual diagnosis among cases of first-episode psychosis) is approximately 15 in 100,000 per year” [2].

The etiology of psychosis varies widely; for the sake of this paper, we will focus on the nutritional and mood associations of the disease. Plenty of research suggests that malnutrition can contribute to psychosis through various pathways. Specific nutritional deficiencies, sleep deprivation, and metabolic derangements are known medical causes of psychosis [3]. It can be argued that eating disorders and psychotic disorders are different expressions of the same illness, with distorted thoughts about eating being a form of delusion [4]. However, instances in which the psychotic event precipitates a metabolic imbalance are much less common in the current medical literature. Recent research has suggested that the comorbidity of eating disorders in mentally ill patients remains relatively unexplored; it has also been reported that schizophrenic and bipolar patients have a statistically significantly higher prevalence of eating disorders than the general population [5].

Antipsychotic drugs, used for the treatment of psychosis, can also affect multiple neurotransmitter systems and exert antagonistic actions on dopamine receptors as well as on serotonergic, histamine, muscarinic, and adrenergic ones. All these neurotransmitters have been directly or indirectly involved in the pathways associated with the regulation of food intake [6].

To our knowledge, very few cases have been reported featuring a psychotic episode that leads to binge eating in an elderly patient with a psychiatric profile significant for mental illness. Here, we present the case of a 71-year-old Hispanic male with a medical history significant for schizoaffective disorder bipolar type who was admitted to the hospital due to acute gastroenteritis preceded by a binge-eating episode alongside a decompensated psychotic state. The patient was medically managed to stabilize his condition and discharged at a psychiatric and biological baseline.

## Case presentation

This is the case of a 71-year-old Hispanic male with a medical history significant for gout, type 2 diabetes mellitus, hyperlipidemia, hypertension, atherosclerotic heart disease, gastroesophageal reflux disease, benign prostatic hyperplasia, and chronic obstructive pulmonary disease, and a psychiatric medical history significant for schizoaffective disorder bipolar type. He was initially admitted to the medical floor due to the acute presentation of gastroenteritis which had been preceded by a binge-eating event at his assisted living facility (ALF). The patient recalled having various similar episodes in the past with the last one occurring about three months ago but none precipitating gastroenteritis. He was referred to the psychiatric department because while he admitted to not being physically hungry, he continued to eat even when uncomfortably full. He was also exhibiting abnormal hostility and aggression toward the ALF staff, all the while demanding more food, despite having already consumed excessive amounts thereof. Once his acute gastroenteritis was stabilized, the patient was admitted to the psychiatry department. Upon arrival, he needed intensive medical management to stabilize his mental status.

On the first day of examination, the patient appeared anxious and disheveled. He exhibited a manic mood featuring psychomotor agitation, impaired attention and concentration, flight of ideas, decreased need for sleep, and disorganized speech, as evidenced by tangential associations, although easily redirected. He also presented with abnormally fast, loud, and repetitive speech. He was alert and oriented to person and place but had no insight into his condition. He was unable to calculate serial 7s or recite all months from the year backward. His thought process appeared to be tangential, although easily redirected. His recent memory was impaired, as evidenced by the inability to recall his last meal (consumed one hour prior). However, regarding remote memory, he successfully recalled his date and place of birth. He maintained poor eye contact throughout the interview, and simple math skills were also impaired. An initial Mini-Mental State Exam (MMSE) score of 19 was obtained.

On physical examination, the patient’s vital signs were within normal limits. His lungs had coarse breath sounds bilaterally with mild expiratory wheezes without rales or crackles. His abdomen was soft and non-tender, bowel sounds were present, and his heart sounds presented normal S1/S2, mildly tachycardic. Strength was 5/5 bilaterally in the upper and lower extremities. He presented with tardive dyskinesia and echolalia (repeating almost every word said to him with little insight). Abstraction was personalized, evidenced by impaired interpretation of proverbs. The patient was clearly responding to internal stimuli and admitted to having auditory and visual hallucinations, with the former being commanding voices telling him to kill himself and the latter manifesting as indistinguishable shadows. Of note, the patient emphasized not wanting to harm himself and had a strong desire to ignore the commanding voices. The patient denied drug use, and it was affirmatively confirmed through a drug screen panel. His gait was unsteady, but he could ambulate without assistance.

Upon arrival at the psychiatric department, the patient’s home medications included ziprasidone 20 mg twice a day in combination with quetiapine 200 mg qd, buspirone 10 mg bid, and Depakote (valproic acid) 250 mg bid. His valproic acid level, however, was at 13.0 µg/mL (therapeutic range 50-100 µg/mL) on admission. To stabilize the patient’s current condition, we discontinued quetiapine and increased the ziprasidone dose to 80 mg PO at bedtime. Buspirone was discontinued, and lorazepam 1 mg twice and temazepam 15 mg at bedtime were ordered instead. The valproic acid dose was increased to 500 mg every eight hours, and benztropine 1 mg bid was added to his medications. The patient was initially diagnosed with an acute exacerbation of schizoaffective disorder bipolar type with multiple episodes. His Young Mania Rating Scale score was 31/60, indicating a moderately manic presentation. In response to the prompt medical management of his condition, he rapidly and safely returned to baseline functioning.

Within 24 hours of medication adjustment, on bedside examination, the patient exhibited a manic mood accompanied by psychomotor agitation. His tardive dyskinesia had been significantly reduced, and his echolalia and disorganized speech also showed symptoms of improvement, as evidenced by a more paused, alas, still fast speech and involuntary repetition of only one of every three or so phrases said to him. The patient was alert and oriented to person and place but had no insight into his condition. He had minor signs of neurocognitive impairment but not as severe as they were on admission.

Within 48 hours of medication adjustment, the patient no longer exhibited a manic mood, and he was calm and collected. No psychomotor agitation was noted, and his tardive dyskinesia, echolalia, and disorganized speech had completely resolved. His repeat Young Mania Rating Score was 10, indicating remission of his manic state, and his MMSE score was 26 indicating significantly improved mental status compared to his initial presentation. The patient was alert and oriented to person, place, time, and situation and evidenced insight into his condition. He maintained good eye contact, was able to maintain a conversation appropriately, and his recent memory and simple math skills were intact. His affect was full and appropriate, and his thought process appeared to be goal-oriented. Individual supportive psychotherapy was provided to assure compliance with medication, and the patient was advised regarding adverse effects associated with the use of psychoactive drugs such as neuroleptic malignant syndrome, photosensitivity, heat sensitivity, extrapyramidal symptoms (EPS), and serotonin syndrome.

## Discussion

When treating a decompensated psychiatric patient, like the one presented here, it is necessary to take a holistic approach to address all the patient’s issues. Our patient’s main psychiatric problems included acute psychosis, moderate mania, and tardive dyskinesia. We will now briefly review each of these disorders as pertaining to our case.

Manic episodes and psychotic features

The aggression and agitation our patient exhibited upon presentation are features found in both psychotic and manic episodes. While psychosis may present with various signs, in approximately 20% of clinical manifestations, we can find agitation/aggression [7,8]: a state of acute anxiety and heightened emotional arousal with increased motor activity [9]. Of note, psychotic features in the setting of mania are associated with greater symptom severity and higher morbidity in the long term [10]. Stabilization and appropriate management of this case was crucial for the benefit of our patient. The management of psychosis is heavily dependent upon the etiology of its presentation. Antipsychotic medications are the gold-standard treatment for psychotic episodes and disorders, and the choice, dosing, and administration of the medication will largely depend on the scenario [6]. Upon admission, our patient had been previously prescribed two second-generation antipsychotics. However, given they were both of the same generation and this carries a risk of developing extrapyramidal side effects, as discussed later, we decided to discontinue quetiapine and increase the ziprasidone dose. We chose ziprasidone over quetiapine because the latter carries a risk of exacerbating the stimulation of muscarinic and serotonergic receptors which ultimately cause increased hunger [11], thus exacerbating the patient’s symptoms. Quetiapine 200 mg qd was discontinued and ziprasidone 80 mg PO was ordered.

Disorganized speech and excited catatonia

Catatonia, a syndrome often associated with schizophrenia and psychosis, manifests as an inability to move or behave normally due to an altered mental status. It may also involve repetitive or catatonic excitement (e.g., excessive, purposeless motor activity). Negative symptoms include expressiveness, apathy, flat affect, and anergia. These symptoms may be secondary to other manifestations of the illness (i.e., depression) or due to antipsychotic medications (EPS). Catatonia is a behavioral syndrome marked by an inability to move normally and can occur in patients with underlying medical and psychiatric disorders, particularly schizophrenia. Among the subtypes of catatonia, we can find the excited subtype, which is characterized by excessive and purposeless motor activity, restlessness, echolalia, stereotypy, impulsivity, frenzy, agitation, and combativeness [12].

Echolalia is the unsolicited repetition of utterances made by others. It is one of the most common echo phenomena and is a non-voluntary, automatic, and effortless pervasive behavior. It has been observed in various mental disturbances, including but not limited to delirium, dementia, catatonia, and others [12,13]. Upon admission, our patient’s speech was not well articulated, significantly fast and pressured, and he was difficult to understand and interrupt. He answered nearly every question by quickly repeating said question word by word regardless of the question’s length and subsequently answering in a congruent manner. This presentation, alongside the excessive motor activity, impulsivity, and echolalia, indicates that the patient may have been in an excited catatonic state. Catatonic excitement and impulsivity are most safely treated with a benzodiazepine such as lorazepam, although seclusion and physical restraints are occasionally required as well [12,13]. To this end, and to address the patient’s insomnia and acute agitation, the use of lorazepam 1 mg bid and temazepam 30 mg at bedtime was warranted. Due to its aggressive presentation, failure to stabilize this aspect of the patient’s condition could have endangered his safety and that of others.

Tardive dyskinesia

Tardive dyskinesia is a movement disorder resulting from treatment with typical and atypical antipsychotics. An estimated 16-50% of patients treated with antipsychotics have tardive dyskinesia, but this number may be underestimated [14]. Our patient initially presented with involuntary movements involving his facial muscles and mouth, as evidenced by involuntary chewing motions and lip-smacking; effects theorized to be due to chronic EPS brought on using two atypical antipsychotic medications in tandem at a dose that was not significant enough to control the patient’s psychosis but did provoke the undesired side effects (i.e., EPS, tardive dyskinesia, etc.). As aforementioned, to best assist our patient, we discontinued the quetiapine and increased the ziprasidone dosage from 20 mg to 80 mg. The change rapidly resolved the EPS while also stabilizing the patient’s psychotic state.

Psychomotor agitation

Psychomotor agitation may be evidenced by increased motor activity (e.g., excessive gesturing) and emotional activation but may also be accompanied by emotional lability and a decreased level of attention and alterations in cognitive function [9,15,16], all of which were observed in our patient, as evidenced in the case presentation. Psychomotor agitation is particularly prevalent among the schizophrenia and bipolar disorder population [15]. To address psychomotor agitation, we decided to discontinue buspirone, a drug commonly used to treat anxiety disorders, and ordered lorazepam 1 mg twice and temazepam 15 mg at bedtime. In the case of psychiatric agitation, the preferred pharmacological treatment option, if agitation is due to psychotic symptoms, is antipsychotic agents, although benzodiazepines may also be considered when agitation is due to a non-psychotic agitation [15,16]. Because we had already increased the dose of ziprasidone (antipsychotic) and discontinued quetiapine (antipsychotic), the addition of benzodiazepines was warranted to help stabilize the patient’s acute condition.

Binge eating in psychosis

Eating disorders are disabling, deadly, and costly mental disorders that considerably impair physical health and disrupt psychosocial functioning. Estimates are that yearly over 3.3 million healthy life years worldwide are lost because of eating disorders [17]. One study evaluated the symptoms of binge-eating disorder among 31 patients with schizophrenia. In this group, three out of the five patients who met the criteria for binge-eating disorder reported the onset of signs after treatment with atypical antipsychotics [11]. More recently, a different study showed a 6% prevalence of binge-eating disorder among 68 patients with schizophrenia and bipolar disorder [18]. When the etiology of eating disorders is complicated by psychosis, as in this case, prompt management of the psychotic features leading to these binge-eating episodes becomes vital to the preservation of the patient’s physical and mental health. While the frequency of binge-eating episodes in our patient was not sufficient to diagnose a binge-eating disorder, which requires consuming large amounts of food in a short time period, at once a week for three months, he did have a history of frequent binge-eating episodes, with the last known episode being about three months prior to admission. This raises the question of whether said frequency could increase and evolve into a binge-eating disorder if the patient remains untreated. Upon admission, our patient had a urine osmolality of 261 mOsm/kg (normal: 500 to 850 mOsm/kg) and a sodium level of 131 mEq/L (normal: 135-145 mEq/L) theorized to have been brought on by his binge-eating episode. The physical implications of hyponatremia and hypoosmolality have been discussed at large over the years and are most undoubtedly life-threatening [19]. In the setting of underlying psychosis, appropriate maintenance therapy with antipsychotic medication is warranted to prevent further exacerbations of the patient’s mental illness, which may manifest as these life-threatening binge-eating episodes. In the case of our patient, adjusting his medications was sufficient to safely and promptly stabilize his condition and maintain him at baseline functioning throughout the course of his hospitalization.

## Conclusions

Psychosis is a complex and debilitating illness that often impairs the social roles of individuals and their functionality within our society. Furthermore, it is a disease capable of endangering the physical health of an individual; if not adequately treated, it may become life-threatening. Due to the broad and varying manifestations of the disease, it is a clinical challenge to identify the correct course of treatment that will best serve our patients. As evidenced by this case, misuse of medications and inappropriate dosages often exacerbate the symptoms we aim to prevent while causing the patient additional harm. Further research must be done on the efficient management and treatment of this disease. With this case report, we aim to set a precedent describing a rare presentation of psychosis complicated by binge eating, mania, psychomotor agitation, tardive dyskinesia, and disorganized speech, where the psychotic episode was found to be the trigger for a binge-eating episode leading to acute gastroenteritis and hyponatremia. While the acutely ill presentation of an elderly patient with underlying psychiatric illness and various comorbidities posed a clinical challenge, titrated medication adjustments and prompt intervention were sufficient to stabilize the patient’s altered mental status and return him to a psychologically functional state.
